# Deaths among adults under supervision of the England and Wales’ probation services: variation in individual and criminal justice-related factors by cause of death

**DOI:** 10.1186/s40352-024-00263-y

**Published:** 2024-02-27

**Authors:** Karen Slade, Lucy Justice, Frederica Martijn, Rohan Borschmann, Thom Baguley

**Affiliations:** 1 School of Social Sciences, Department of Psychology, Nottingham Trent University, Nottingham, NG1 4FQ UK; 2 Centre for Mental Health, Melbourne School of Population and Global Health, University of Melbourne, Melbourne, VIC 3052 Australia; 3 Department of Psychiatry, University of Oxford, Warneford Hospital, Oxford, OX3 7JX UK; 4 Centre for Adolescent Health, Murdoch Children’s Research Institute, Melbourne, VIC 3010 Australia; 5 Melbourne School of Psychological Sciences, The University of Melbourne, Melbourne, VIC 3052 Australia; 6 School of Population Health, Curtin University, Perth, WA Australia

**Keywords:** Probation, Death, Prison, Suicide, Drug-related death, Homicide, Violence

## Abstract

**Background:**

The mortality rate among people under probation supervision in the community is greater than that among incarcerated people and that among the general population. However, there is limited research on the distinct vulnerabilities and risks underlying the causes of death in this population. In this retrospective cohort study, we examined the individual and criminal justice-related factors associated with different causes of death. Factors were assessed in relation to the type of supervision, distinguishing between those under post-custodial release and those serving a community sentence.

**Results:**

The study utilised the official data held by His Majesty’s Prison and Probation Service in England and Wales on the deaths of men and women under probation supervision between 01 April 2019 and 31 March 2021 where the cause of death had been definitively recorded (*n* = 1770). The high risk of deaths primarily caused by external factors (i.e., suspected suicide (10%), homicide (5%), and drug-related death (26%)) in this population was confirmed. A Gaussian Graphical Model (GGM) demonstrated unique relationships with suspected suicide and drug-related deaths for known suicide risk, history of drug use and recent (< 28 days of death) enforcement action due to a breach of probation conditions. Our findings suggest that that familial violence and abuse may be relevant in suicide and drug-related deaths and that minority groups may experience disproportional risk to certain types of death.

**Conclusions:**

This study identified unique risk indicators and modifiable factors for deaths primarily caused by external factors in this population within the health and justice spheres. It emphasised the importance of addressing health inequalities in this population and improved joint-working across health and justice. This involves ensuring that research, policies, training, and services are responsive to the complex needs of those under probation supervision, including those serving community sentences. Only then can we hope to see lower rates of death within this population.

**Supplementary Information:**

The online version contains supplementary material available at 10.1186/s40352-024-00263-y.

## Introduction

People under probation supervision are at increased risk of premature mortality when compared to both the general and incarcerated populations (Gelsthorpe et al., 2010; Sattar, 2001; Skinner & Farrington, 2020; Wildeman et al., 2019; Zlodre & Fazel, 2012). Recent years have seen the development of an extensive research body on premature and non-natural deaths among people in custodial settings, including suicide and drug-related deaths, and a subsequent increase in efforts to reduce the numbers of preventable deaths in custody (Equality & Human Rights Commission, 2015; Favril et al., 2020; Harris, 2015; World Health Organization, 2014). However, people who die after they are released from custody and/or under community sentence have received significantly less research and policy attention than people who die whilst in custody (Phillips et al., 2019b), despite making up the majority of deaths in justice-involved populations (Ministry of Justice, 2022a). This paucity of research has likely impeded meaningful progress towards identifying candidate interventions to prevent these deaths and improve health outcomes in this marginalised population.

In England and Wales (E&W), the criminal justice system (CJS) consists of the police, the Crown Prosecution Service, the courts, HM Prison and Probation Service (HMPPS) and the Youth Justice Board, the latter two of which are is overseen by the Ministry of Justice. On conviction, people may be sentenced to a community sentence (a sentence which combines some form of punishment with activities carried out in the community) or they may be sentenced to a custodial sentence (imprisonment). Almost all people either released from custody or receiving a community sentence within E&W will be subject to supervision by the Probation Service (Her Majesty’s Inspectorate of Probation (HMIP), 2020). The number of people under community supervision is around three times as many as those in custody; in E&W in March 2022, there were 172,492 people under community supervision, and 80,859 were serving custodial sentences (Ministry of Justice, 2022a). Amongst those under probation supervision 88% are male and 17% of the population were over 50 years of age (Cadet, 2020).

The overall rates of deaths of people under supervision in the community have continued to increase, with over 1000 deaths reported in E&W annually since 2018/9 (Ministry of Justice, 2022b), compared with approximately 350 deaths in custody over the same period (Ministry of Justice, 2022c). There have been growing calls to gain a better understanding of the risks of mortality for people under community supervision (Phillips et al., 2019a, b; Sattar, 2001). However, few jurisdictions publish official figures for deaths whilst under supervision, with E&W being one of the few to do so since 2011 (Ministry of Justice, 2022b). The limits on collated or published data contribute substantially to the paucity of research and understanding of this population.

Crucially for prevention and policy, causes of mortality amongst people under supervision are starkly different from those in the general population. People under community supervision have an overall three-times elevated standardised mortality ratio (SMR; i.e., their rate of death is three times higher than that of their peers from the general population) (Sattar, 2001) with a higher rate of suicide and drug-related deaths than reported in prison custody (Laine et al., 2022; ONS, 2023). Deaths by natural causes are often defined to be deaths whose primary cause was the progression of disease or illness, e.g., cancer or heart disease and are the most common cause of death in the general population (over 90% of deaths; Office for National Statistics (ONS), 2022a). Conversely, within the population under supervision, cases categorised as natural causes only account for 20–50% of deaths (Gelsthorpe et al., 2010; Phillips et al., 2019a; Sattar, 2001) although they are often contributed to by external factors such as substance use and inadequate poor healthcare provision (Shapiro & Keel, 2023; Woudenberg-van den Broek et al., 2022).

The increased risk of death primarily caused by these external factors such as intentional harm, substance use, violence or accident amongst the population under supervision is consistent across studies and countries (e.g., Australia, France, Sweden, the UK, and the USA), and demonstrate an considerably elevated risk of dying by drug overdose (e.g., Farrell & Marsden, 2008; Merrall et al., 2010), suicide (e.g., Jones & Maynard, 2013; Pratt et al., 2006), accidents (e.g., Binswanger et al., 2007), and homicide (e.g., Willoughby et al., 2021; Zlodre & Fazel, 2012). Deaths occurring under probation supervision have been largely studied amongst the post-custody release population, and sparingly amongst the community sentence population, even though community sentences play a central role in many criminal justice systems (Heard, 2015; Phillips et al., 2019b).

Several risk groups are overrepresented among people who are in contact with the CJS, compared with the general population, which may contribute to an increased likelihood of premature death. Men are disproportionately in contact with the CJS with 88% of those under probation supervision being men (Ministry of Justice, 2022a), and men in the general population are at three times higher risk than women to die from both homicide and suicide (Office for National Statistics, 2022b). Other known risk factors for premature deaths include unmet physical and mental health needs (Brooker et al., 2014; Sirdifield et al., 2020a), poverty, family conflict, violent offending (Guy, 2010; Zhong et al., 2021), and substance misuse issues (Her Majesty’s Inspectorate of Probation, 2020; Sirdifield et al., 2020b). Drug and alcohol misuse has been identified in around half of people under probation supervision (Her Majesty’s Inspectorate of Probation, 2020). Although few studies have been explored the mental health needs for those under supervision, there is evidence that between 25–38% meet the criteria for a mental health disorder (Brooker et al., 2014; Sirdifield et al., 2020a). Furthermore, disproportionality identified in mortality rates for people with identity characteristics known as ‘protected characteristics’ (i.e. protected from discrimination on the basis of these characteristics in UK law) including ethnic background (e.g., suicide, Office for National Statistics, 2021) or religion (e.g., homicide, Litvak et al., 2023) are yet to be explored for this high-risk populations.

The social and structural drivers of criminal justice system involvement overlap to a considerable degree with the drivers of poor health, and of preventable morbidity and mortality (Borschmann et al., 2020) As such, many people who experience incarceration have poor health profiles and are at an increased risk of early mortality. There is an acute elevation of mortality risk during post-custody release, particularly in the first month after release (Binswanger et al., 2007; Farrell & Marsden, 2008; Haglund et al., 2014; Kinner et al., 2013; Merrall et al., 2010; Pratt et al., 2006; Ranapurwala et al., 2022), with women especially at-risk during this period (Farrell & Marsden, 2008; Gan et al., 2021). Around 44,000–64,000 people are released from custody in E&W each year (Ministry of Justice, 2022d) with many facing further difficulties during the return to the community which are associated with premature mortality e.g., facing homelessness or temporary housing, debts, difficulties in accessing healthcare, and unemployment (Gelsthorpe et al., 2010; Social Exclusion Unit, 2002). However, pre-existing risk factors do not wholly explain the acute elevation of mortality risk in this population. Therefore, being under community-based supervision by probation may itself increase the risk of mortality, for instance through elevated stress levels (Kinner et al., 2013) with previous incarceration shown to have long-lasting effects on health outcomes (Schnittker & John, 2007). Evidence is also emerging that actions undertaken by the CJS may act as risk markers for suicide, such as when the Probation Service initiated action following a breach or violation of community-based supervision requirements (known as ‘enforcement action’) (Borrill et al., 2017; King et al., 2015).

The community sentence population are demographically largely similar to those under post-custody release, but meaningful differences in mortality risk may be obscured when studies combine, instead of compare, these populations as “the probation population” (e.g., Biles et al., 1999; Pritchard et al., 1997; Sirdifield et al., 2020a, b). There are potential distinctions between these groups, such as the situationally different stressors of the custody-to-community transition versus serving a sentence while maintaining, to some degree, the status quo of one’s life in the community (Phillips et al., 2019b). A better understanding of both the unique and overlapping needs and risks of both these populations is necessary to develop an appropriate approach to preventable deaths.

This paper focuses on people in E&W who were under community supervision by the Probation Services at the time of their death, either post-custody or whilst serving a community sentence. The current study aims to document the causes of death and the individual and criminal justice-related factors associated with different causes of death. The study will focus on deaths primarily caused by external factors (i.e., drug-related deaths, suicide, accidents, and homicide) and include comparisons based on supervision type (i.e., those on post-custody release versus those serving a community sentence).

Specifically, this study will explore: whether there is an overrepresentation of deaths primarily caused by external factors (i.e. suspected suicide, homicide, drug-related deaths and accidents) compared with deaths primarily categorised as natural cause deaths within this population; whether specific individual or criminal justice-related variables are uniquely related to different causes of death within a Gaussian Graphical Model (a network that displays partial correlations between pairs of variables from the full set); and which factors uniquely associate with a cause of death, within a Gaussian Graphical Model, and hence whether there are different unique partial correlations based on type of sentence (post-custody release versus a community sentence).

## Method

### Study population

This study is a retrospective cohort study, analysing the records of all people whose death was recorded while under supervision by HMPPS in E&W between 01 April 2019 and 31 March 2021 (inclusive). During the study period probation services were split between the National Probation Service (NPS; for people deemed to be at high risk of reoffending or serious harm) and Community Rehabilitation Companies (CRCs; for people deemed to be at low or medium risk of reoffending or harm). The data do not include anyone who died during a period of incarceration since their deaths did not occur in the community. The definition of a ‘death under supervision’ refers to those currently supervised by the Probation Services, both on community sentenced and for a period after prison release, as outlined in HMPPS policy (HM Prison and Probation Service and Ministry of Justice, 2022). This study included the period from 1 April 2019 to 31 March 2021 and an increase in the number of deaths classified as natural cause due to the Covid-19 pandemic was expected from March 2020, especially in the older age groups.

### Data source

Data were drawn exclusively from the nDelius electronic case management system where demographic, offence, and other characteristic information is routinely reported by probation staff. All deaths under supervision are reported on the nDelius system based on information provided to the probation practitioner using the definitions outlined in Probation Instruction 01/2014 (Ministry of Justice, 2014) and may not reflect official records e.g. coroner’s outcome.

The HMPPS National Applications Reporting Team extracted the nDelius data and cross-referenced with the Ministry of Justice (MoJ) published data on death under probation supervision with support of the Prison and Probation Analytical Services team. Only data from deaths recorded in the official published data were analysed. Extracted data included dichotomous (e.g., present/absent) and choice-based options for data entry. Some data were recoded into pooled variables to derive the variables.

### Variables

#### Cause of death

HM Prison and Probation Service (HMPPS) uses a classification for apparent causes of death which includes five overarching categories: (1) *Natural causes*: any death primarily due to a naturally occurring disease or illness; (2) *Self-inflicted*: any death of a person who has apparently taken their own life irrespective of intent. Sub-categories for self-inflicted deaths were Drug overdose; Hanging/suffocation; and Other; (3) *Homicides*: any death at the hands of another; (4) *Accident*: any death arising from other external causes [such as deaths resulting from motor vehicle collisions, falls, etc.]; (5) *Unclassified or other*: any death any death that cannot be easily classified or where there is insufficient information to make a judgement about the cause at the time of reporting. Each death is classified based on information at the time of death and reported to HMPPS via the Probation Service. Cause of death classification may be revised following inquest.

All other variables are the last recorded data point in the individual’s record prior to death with labels as per the official data.

#### Characteristics

All characteristics were as recorded by the probation practitioner on the electronic case management system.

*Gender* (*this variable reflects legally recognised gender as per Ministry of Justice* (2024) *and recorded as male/female only*); *Age* (18–24, 25–35, 36–49, 50–65, Over 65), ethnicity (Asian, Black, Mixed and White), and religion (Christian, Muslim, Atheist, No Religion, Other, *Accommodation* (e.g., settled accommodation, inpatient or residential care or homeless); *Employment* (e.g., employed, unemployed, retired); *Sentence Type*: Post-custody or community sentence; *Offence Type*: Data regarding most recent offences were condensed into superordinate categories (categorisations as outlined in Howard et al., 2009) and used the offence(s) recorded at the most recent sentencing occasion (Violence against the person, Sexual offences, Possession of weapons, Fraud, Theft, Public Order Offences, Drug Offences, Robbery, Summary Motoring, Miscellaneous); *Enforcement Action* (present/absent): enforcement was initiated due to violation of supervision requirements which may trigger a return to court (for community sentences) or a return to custody (for post-custody release) within the final 28 days prior to death; *High Risk of Serious Harm (RoSH)* **(**present/absent): assessed by Probation as a high risk of harm to others.

The following variable was reported by the probation practitioner as being known risks on nDelius case management system as active/not active within 12 months of death. *Drug Misuse*: Recorded as having a drug testing requirement or evidence of drug misuse.

The following variables are the presence/absence of the specific factor using the labels assigned in the data. These are based on the subjective assessment of the probation practitioner and not structured tools or diagnostic criteria, although may be informed by information received from other agencies. *Suicide or Self-harm risk; Mental health problems; Domestic violence (DV) perpetration; Domestic violence (DV) victimisation*. The Domestic Violence (DV) label includes a broad definition covering both intimate partner violence and abuse and of other family members.

### Data analysis

First, simple descriptive and comparative statistics are presented, using ANOVA and Chi-square analyses in combination with post-hoc tests corrected with the False Discovery rate.

Second, as variables related to an increased risk of death are rarely singular and often interrelate, to facilitate exploratory network analysis, a Gaussian Graphical Model (GGM; Epskamp & Fried, 2018) analysis was performed to examine the unique partial relationships between the variables and the causes of accidental, drug overdose, suspected suicide, and homicide deaths when compared with deaths classified as natural causes. GGMs were estimated using the Bayesian BGGM package in R (Williams & Mulder, 2020), which can handle binary, ordinal, and continuous data and impute missing data during model fitting. GGM is used to depict partial correlations between pairs of nodes (variables represented as circles) and edges (lines indicating the strength and direction of partial correlations). GGM is an exploratory approach which examines the unique partial correlation (i.e., the correlation between two variables while removing the correlation with all other variables in the model). This represents the contribution of that bivariate association over and above the indirect impacts of variables in the full set. Positive relationships are reported in green and negative relationships in orange, with thicker lines representing stronger associations. The partial correlations remove (partial out) the contribution of other nodes in the network. Thus, they show the unique association between variables. This can be particularly valuable where there are many intercorrelated variables which make interpretation of the simple correlations challenging. Partial correlations including zero in the 95% (90% for sub-groups) posterior probability intervals (where the model estimates the true association lies, with 95% (or 90%) probability) are not shown in the plot, and hence only the strongest relationships are retained in the graph (Epskamp & Fried, 2018).

## Results

### All-cause mortality

A total of 2448 deaths under supervision were recorded during the study period between April 2019 and March 2021 [April 2019 to March 2020 = 999; Apr 2020 to March 2021 = 1449]. The average annual number of people under community probation supervision in E&W during this period was 168,914 (Ministry of Justice, 2022a).

The average age at the time of death was 45.1 (*SD* = 14.8) years, and most decedents were male (87.3%) and White (92.3%). In terms of supervision status 49.1% were on post-custody release, and 50.9% were serving a community sentence. Of the 2448 deaths under supervision recorded, 748 were excluded from the sample. Two cause of death categories were excluded because the specific cause of death was unspecified or still under investigation: ‘Awaiting further information’ (*n* = 564) and ‘Self-inflicted: Other or Unspecified’ (*n* = 184). Based on findings from Office for National Statistics (ONS) (2023), these categories would include an amalgamation of other causes of death including other forms of suspected suicide, fatal self-harm and alcohol-related deaths as well as currently undetermined drug-related deaths. To maintain data integrity, a total of 1700 people were included in the sample.

The 1700 included cases were compared with the 748 excluded cases for any differences, using Chi-square analysis. There were no significant differences in sex or ethnicity between the groups. Significantly more individuals on post-custody release were in the included sample (${X}^{2}$ (1, 2447) = 20.4, *p* < 0.001; 57.8 vs 42.2%). Analysis provided in Additional file 1 provides further detail.

### Included sample

#### Causes of death

A total of 51.4% (*n* = 873) were classified as having died primarily from natural causes, with 48.6% (*n* = 827) classified as deaths caused primarily by external causes: 25.9% (*n* = 440) of drug overdose; 10.0% (*n* = 170) by suspected suicide (encompassing reported methods of hanging, suffocation, or intentional fall from height); 7.9% (*n* = 135) through accidental death; and 4.8% (*n* = 82) from homicide. Details of official MoJ death categorisations are available in Ministry of Justice (2022b). Based on the average annual rate of people under supervision, rates for drug overdose were calculated as 130 per 100,000 persons, suspected suicides as 50 per 100,000, accidental death as 40 per 100,000 and homicide as 24 per 100,000.

### Sample descriptive

The demographic details for the sample are outlined in Table 1.

**Table 1:** Number, percentage and inferential test (ANOVA or Chi-square) of each variable by cause of death

		**All**	**Natural cause**	**Drug overdose**	**Suicide**	**Accident**	**Homicide**		
		**(*n* = 1700)**	**(*n* = 873)**	**(*n* = 440)**	**(*n* = 170)**	**(*n* = 135)**	**(*n* = 82)**		
**Demographic information**		*M* (*SD*)	*M* (*SD*)	*M* (*SD*)	*M* (*SD*)	*M* (*SD*)	*M* (*SD*)	*df*	*F*
Age (*n* = 1,689)		46.0 (15.6)	54.9 (14.8)_a_	37.6 (8.7)_b_	36.6 (11.0)_bc_	37.6 (8.7)_bcd_	30.2 (9.2)_f_	4, 1685	232.71^***^
		*N* (%)	*n* (%)	*n* (%)	*n* (%)	*n* (%)	*n* (%)	*df*	*χ*^2^
Gender (*n* = 1,700)	Male	1484 (87.3%)	778 (89.1%)	359 (81.6%)^−^	153 (90.0%)	118 (87.4%)	76 (92.7%)	4, 1699	18.72^***^
	Female	216 (12.7%)	95 (10.9%)	81 (18.4%)^+^	17 (10.0%)	17 (12.6%)	6 (7.3%)		
Ethnicity (*n* = 1,663)	Asian	43 (2.6%)	32 (3.8%)^+^	3 (0.7%)^−^	1 (0.6%)	2 (1.5%)	5 (6.2%)	12, 1651	123.73^***^
	Black	53 (3.2%)	26 (3.1%)	6 (1.4%)	3 (1.8%)	4 (3.0%)	14 (17.5%)^+^		
	Mixed	32 (1.9%)	7 (0.8%)^−^	9 (2.1%)	4 (2.4%)	3 (2.3%)	9 (11.2%)^+^		
	White	1535 (92.3%)	787 (92.4%)	415 (95.8%)	157 (95.2%)	124 (93.2%)	52 (65.0%)^_^		
Religion (*n* = 1,174)	Christian	343 (29.2%)	184 (32.1%)	88 (27.9%)	30 (25.2%)	29 (27.9%)	12 (19.4%)	16, 1172	100.95^***^
	Muslim	31 (2.6%)	10 (1.7%)	6 (1.9%)	0 (0%)	3 (2.9%)	12 (19.4%)^+^		
	Other	66 (5.6%)	48 (8.4%)^+^	9 (2.9%)	6 (5.0%)	2 (1.9%)	1 (1.6%)		
	No religion	720 (61.3%)	324 (56.4%)^−^	209 (66.3%)	80 (67.2%)	70 (67.3%)	37 (59.7%)		
	Atheist	14 (1.1%)	8 (1.4%)	3 (1.0%)	3 (2.5%)	0 (0%)	0 (0%)		
**Personal circumstances**		*N* (%)	*n* (%)	*n* (%)	*n* (%)	*n* (%)	*n* (%)	*df*	*χ*^2^
Accommodation (*n* = 1,469)	Settled accommodation	1,067 (72.6%)	548 (74.9%)	268 (67.0%)^−^	107 (73.8%)	92 (76.7%)	52 (72.2%)	20, 1469	54.7^***^
	Short-term accommodation	175 (11.9%)	70 (9.6%)	60 (15.0%)	19 (13.1%)	14 (11.7%)	12 (16.7%)		
	Homeless	109 (7.4%)	42 (5.7%)	40 (10.0%)	12 (8.3%)	11 (9.2%)	4 (5.6%)		
	Hospital or residential care	47 (3.2%)	41 (5.6%)^+^	3 (0.8%)^+^	3 (2.1%)	0 (0%)	0 (0%)		
	Probation accommodation	43 (2.9%)	20 (2.7%)	17 (4.2%)	3 (2.1%)	1 (0.8%)	2 (2.8%)		
	Other	28 (1.9%)	11 (1.5%)	12 (3.0%)	1 (0.7%)	2 (1.7%)	2 (2.8%)		
Employment (*n* = 992)	Full time or self-employed	106 (10.7%)	48 (9.8%)	16 (6.1%)^−^	21 (20.8%)^+^	14 (17.1%)	7 (12.7%)	24, 992	150.62^***^
	Parttime (self-) employed (< 30 h),	39 (3.9%)	12 (2.4%)	6 (2.3%)	6 (5.9%)	9 (11.0%) ^+^	6 (10.9%)		
	Unemployed (with financial support)	444 (44.8%)	196 (39.8%)^−^	140 (53.4%)^+^	42 (41.6%)	40 (48.8%)	26 (47.3%)		
	Unemployed (no financial support)	144 (14.5%)	56 (11.4%)	49 (18.7%)	15 (14.9%)	12 (14.6%)	12 (21.8%)		
	Retired	89 (9.0%)	86 (17.5%)^+^	1 (0.4%)^−^	2 (2.0%)^−^	0 (0%)^−^	0 (0%)		
	Unavailable for work	143 (14.4%)	76 (15.4%)	44 (16.8%)	15 (14.9%)	6 (7.3%)	2 (3.6%)		
	Other or unknown	27 (2.7%)	18 (3.7%)	6 (2.3%)	0 (0%)	1 (1.2%)	2 (3.6%)		
**Offence information**		*N* (%)	*n* (%)	*n* (%)	*n* (%)	*n* (%)	*n* (%)	*df*	*χ*^2^
Supervision type	Post-custody release	887 (52.2%)	474 (54.3%)	246 (55.9%)	76 (44.7%)	57 (42.2%)^−^	34 (41.5%)	4, 1696	16.96^**^
	Community sentence	813 (47.8%)	399 (45.7%)	194 (44.1%)	94 (55.3%)	78 (57.8%)^+^	48 (58.5%)		
Enforcement action	Enforcement or recall	294 (17.3%)	115 (13.2%)^−^	100 (22.7%)^+^	35 (20.6%)	25 (18.5%)	19 (23.2%)	4, 1700	22.86^***^
		*N* (%)	*n* (%)	*n* (%)	*n* (%)	*n* (%)	*n* (%)	*df*	*χ*^2^
**Risk factors**	Drug misuse	388 (22.8%)	117 (13.4%)^−^	183 (41.6%)^+^	39 (22.9%)	27 (20.0%)	22 (26.8%)	4, 1700	133.33^***^
	Known suicide risk	315 (18.5%)	108 (12.4%)^−^	105 (23.9%)^+^	59 (34.7%)^+^	29 (21.5%)	14 (17.1%)	4, 1700	60.59^***^
	Mental health condition	959 (56.4%)	282 (32.3%)^−^	229 (52%)^+^	71 (41.8%)	62 (45.9%)	29 (35.4%)	4, 1700	51.14^***^
	High/Very high RoSH	232 (15.8%)	102 (11.7%)	70 (15.9%)	28 (16.5%)	22 (16.3%)	10 (12.2%)	4, 1700	6.87
	DV Perpetration	450 (36%)	180 (20.6%)^−^	140 (31.8%)^+^	62 (36.5%)^+^	44 (32.6%)	24 (29.3%)	4, 1700	33.49^***^
	DV Victimisation	58 (3.4%)	22 (2.5%)	21 (4.8%)	8 (4.7%)	6 (4.4%)	1 (1.2%)	4, 1700	7.08

Percentages indicate percentages within cause of death

Different subscripts indicate significant differences, i.e., if two causes of death have the same subscript (e.g., “b”) there are no significant differences between the causes of death in age. If two causes of death do not share the same subscript (e.g., “a” and “c”), it means the ages of the causes of death differ significantly

Subscript “- “ indicates that a post-hoc test with *p*-values corrected using the False Discovery Rate showed that the number of deaths within this cell is significantly lower than expected within this cause of death

Subscript “ + “ indicates that a post-hoc test with *p*-values corrected using the False Discovery Rate showed that the number of deaths within this cell is significantly higher than expected within this cause of death

*AP* Approved Premises, *BASS* Bail Accommodation and Support Service, *RoSH* Risk of Serious Harm, *DV* Domestic violence

^*^p < .01

^**^p < .01

^***^p < .001

#### Sentencing

Within the sample, 52.2% (*n* = 887) were post-custody releases and 47.8% (*n* = 813) were serving community sentences. Regarding the most recent offence, 28.7% were convicted of violence against the person; 16.0% for theft; 12.8% for sexual offences; 9.6% for public order offences; 8.9% for motoring offences; 6.9% for drug-related offences; the remaining offence types were recorded by less than 5% of the sample. Thirteen individuals had two most recent offences recorded, which are retained in the figures. Cohort members recorded an average of 9.3 sentencing occasions (*SD* = 11.7; Min = 1, Max = 97).

### Differences in demographics and risk factors by cause of death

#### Risk factors by cause of death

A series of Chi-square analyses (for categorical variables) and ANOVA (for age as a continuous variable) were conducted to detect differences in demographics and risk factors by cause of death and hence whether each variable is associated with cause of death. Post hoc analyses was undertaken with *p*-values corrected using the False Discovery Rate (Benjamini & Hochberg, 1995), with full results and comparators outlined in Table 1.

Analysis showed that all variables except the Risk of Serious Harm (RoSH) and domestic violence (or abuse) victimisation showed significant disproportionality for one or more cause of death. The following findings delineate any sub-category that exhibited significant over- or under-representation within each variable category for a cause of death, relative to the expected proportion (i.e. that the listed sub-category (e.g. Asian) had a higher prevalence of that cause of death than expected than if the causes of death were independent of the category type (e.g., ethnicity)).

*Natural causes* deaths were relatively more common within Asian ethnicity, those receiving inpatient or residential care as accommodation status, and unemployed or retired as employment status. They were relatively less common in those with enforcement action within the 28 days preceding death, with a suicide risk, drug misuse or mental health condition or have a history of domestic violence or abuse perpetration. People who died from natural causes were significantly older in age than all other causes of death. *Drug overdose* deaths were relatively more common within female gender, those in short-term accommodation or homeless as accommodation status, unemployed (with financial support), had a recent enforcement action, had risk markers for drug misuse or suicide risk, mental health conditions, or a history of domestic violence (or abuse) perpetration. *Suspected suicide* deaths were relatively more common within those who were fully or self-employed, held settled accommodation, had known suicide risk, or a history of domestic violence (or abuse) perpetration. *Accidental deaths were* more relatively more common in those who were part-time employed or serving a community sentence. *Homicide* deaths were relatively more common in within Black, Mixed or Asian ethnicity or Muslim religion. People who died from homicide were significantly younger in age than other causes of death.

### GGM analysis

The ANOVA and Chi-square analyses identified specific factors as significantly differentiating a cause of death. As deaths classified as natural causes would be used as the comparator for this analysis, age was excluded since the proportion of these deaths were heavily skewed by the Covid-19 pandemic. All factors were included in the analysis as a dichotomous variable with one category reported as ‘present’ and all other categories pooled as ‘absent’. Since there may be differential factors for those leaving custody and those on community sentences, analyses were completed separately for these sub-samples.

The models present data relative to deaths classified as primarily due to natural causes as the reference category. Thus, a positive association between a cause of death and another variable indicates both an elevated association relative to death classified as natural causes and a negative association indicates a decreased association. Furthermore, it presents only those factors which retain a unique association after partialing out the impact of other variables in the network.

The full partial correlations table can be found in the Additional file 2. GGM plots provide a rich exploratory tool for handling inter-correlated data and should be interpreted with care, but some potentially important patterns emerge. As the data do not have two causes of death for any individual, all causes of death are negatively correlated with each other. Paths where the 95% posterior probability interval includes zero are not depicted. Positive associations are in green, and negative associations are in orange, with thicker lines indicating stronger partial correlations.

### Full sample

**Fig. 1:**
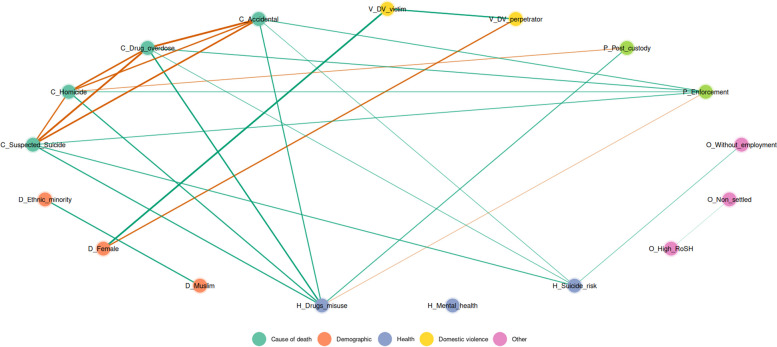
Gaussian graphical model of the partial correlation network for full sample causes of death

Figure 1 shows the GGM for the full included sample. The following unique associations between variables were identified for the full sample: *Drug misuse*: Positive associations were identified with all causes of death (*r* between 0.514 and 0.647). *Suicide/self-harm risk*: Strong associations were identified with deaths primarily from external causes: Drug overdose (*r* = 0.334465), suspected suicide (*r* = 0.461), and accidental deaths (*r* = 0.325). Having a known suicide/self-harm risk was also associated with unemployment, *r* = 0.308. Unemployment was not directly linked to any cause of death. *Enforcement*: Enforcement action was positively associated to all four external causes of death, with *r* values between 0.363 and 0.437. Enforcement action was also negatively related to known drug misuse, *r* = -0.271. *Post-custody release*: Those who were on post-custody supervision were negatively associated with death by homicide (*r* = -0.369), and positively related to having known drug misuse (*r* = 0.443).

### Post-release

**Fig. 2:**
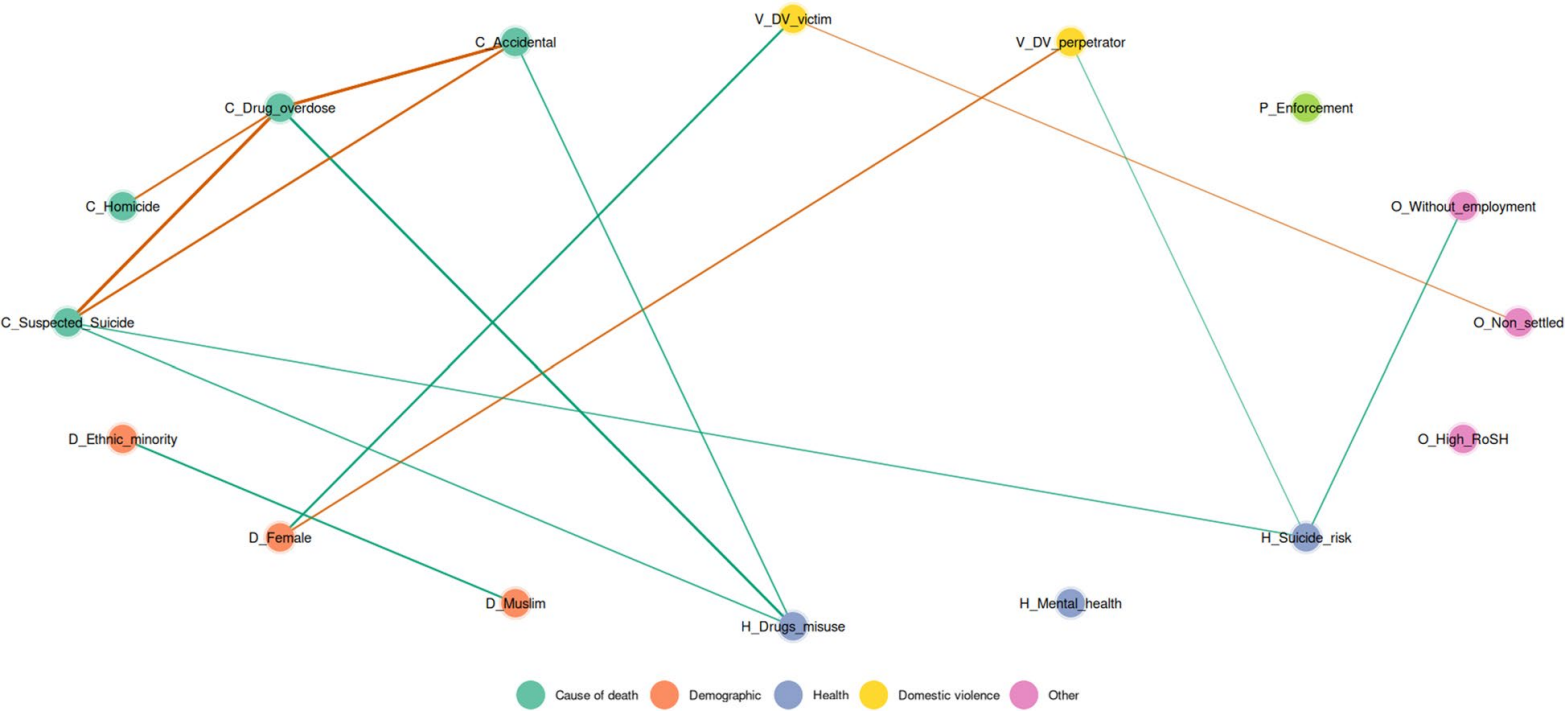
Gaussian graphical model of the partial correlation network for the post-custody release population

A 90% CrL GGM was performed with the same variables only with the post-custody release sample (Fig. 2). The following unique associations between variables were identified: *Drug misuse* was related to drug overdose (*r* = 0.585), suspected suicide (*r* = 0.392), and accidental deaths (*r* = 0.401). *Suicide/self-harm risk* was related to suspected suicide (*r* = 0.364), as well as unemployment (*r* = 0.385) and domestic violence perpetration (*r* = 0.287). Neither unemployment nor domestic violence were directly linked to any cause of death.

#### Community sentence

**Fig. 3:**
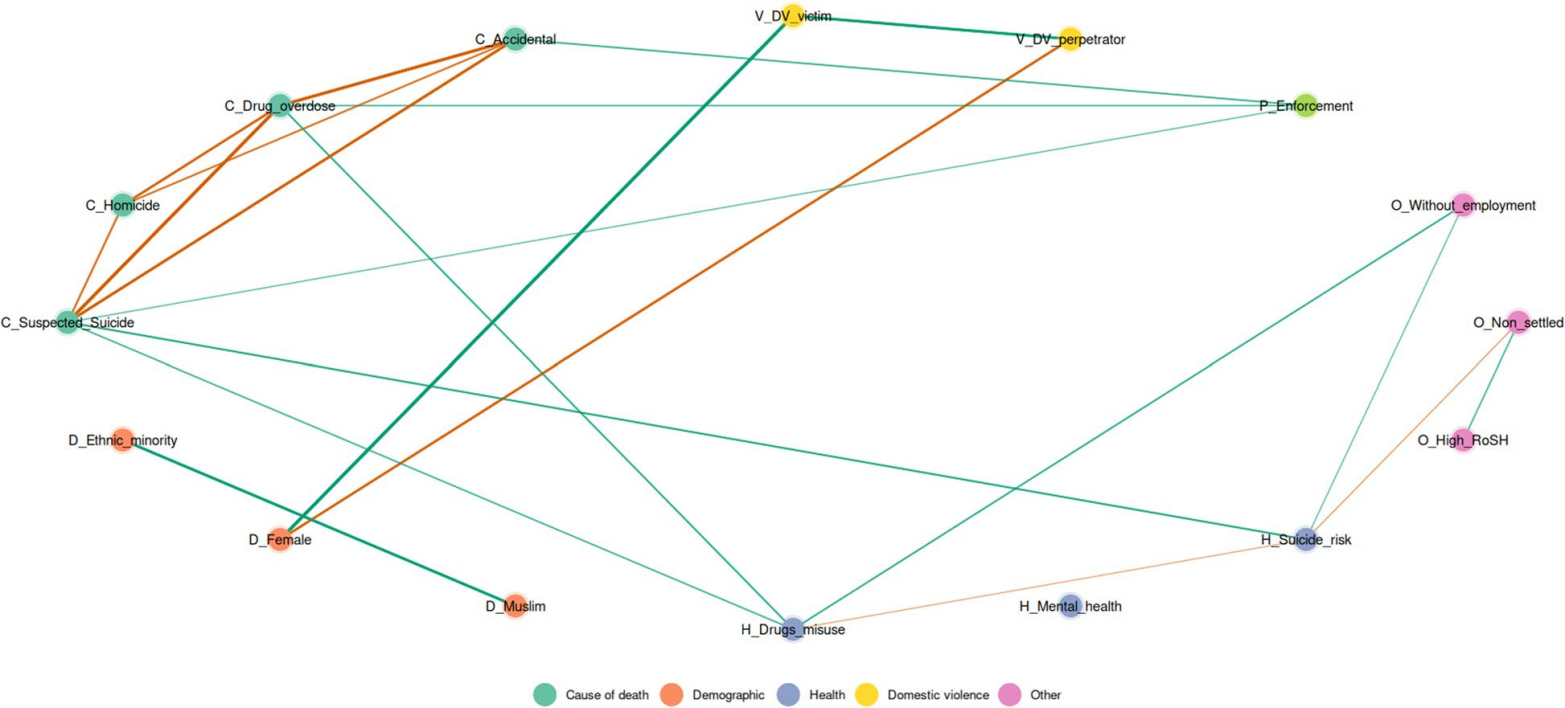
Gaussian graphical model of the partial correlation network for the community sentence population

A GGM was performed with the same variables with the community sentence population (Fig. 3). The following unique associations between variables and causes of death and key risks were identified: *Drug misuse* was associated with deaths by drug overdose (*r* = 0.433), suspected suicide (*r* = 0.377) and unemployment (*r* = 0.421) but negatively related with known suicide risks (*r* = -0.263). *Suicide self-harm risk* had a strong association with deaths by suspected suicide (*r* = 0.461) and unemployment (*r* = 0.298) and negatively with non-settled accommodation (*r* = -0.286). Neither unemployment nor accommodation status were directly linked to any cause of death *Enforcement* was associated with death by drug overdose (*r* = 0.369), suspected suicide (*r* = 0.309), and accidental deaths (*r* = 0.396).

## Discussion

The study aimed, using official secondary data, to detail the prevalence by cause of death for those under probation supervision in England and Wales and document individual and criminal justice-related factors associated with different causes of death. This was the first study to demonstrate how key risk factors relate to specific causes of deaths classified as primarily caused by external factors (drug-related, suspected suicide, accidents, and homicides) when compared with deaths classified as primarily from natural causes, and report differences in these factors based on supervision type (i.e., those on post-custody release versus those serving a community sentence).

This study identified a very high proportion of deaths classified as primarily caused by external factors in this population. Where a cause of death was classified, 26% of cases were reported as drug overdose (130 per 100,000 persons under community supervision annually), 10% as suspected suicides (50 per 100,000), 8% as accidental death (40 per 100,000) and 5% as homicide (24 per 100,000). These rates are likely an underestimate of prevalence because of the excluded deaths and even though there are definitional differences, these rates are much higher than in the general population e.g., Office of National Statistics (ONS) report annual population death rates of around 1 in 100,000 from homicide, 10 per 100,000 from suicide, and 8 per 100,000 from drug poisoning. These deaths account for nearly 50% overall and reflect a hugely elevated risk of premature and death from external causes in this population that has significant public health implications.

Within the multi-factorial network analysis for the full sample, there were three factors, drug misuse, suicide or self-harm risk and enforcement action which were directly related to suspected suicide, drug-related and accidental deaths. Indirect relationships to these types of death, potentially mediated by these factors, was reported concerning unemployment and the perpetration of familial violence. For both post-custody and community sentences, there was clear prominence of the unique contributions of both known drug misuse and suicide risk on the risk of deaths with consistent associations with drug overdose, suspected suicide, and accidental deaths. Suicide or self-harm risk had been identified by probation in 33% of suspected suicides but also 24% of drug-related deaths. This reflects consistent evidence of the interlink between drug use and suicide e.g., drug misuse as a risk factor for suicide, with one in five suicides in the general population being from drug poisoning (Office for National Statistics, 2022a) rising to 45% within cohorts of people with substance misuse issues (Oyefeso et al., 1999). The interlinking nature of these factors highlights their need for prominence in all public health and service interventions in the prevention of premature death. The scale of risk in probation populations reinforces the need for partnership working since these individuals will likely be in contact with both justice and health and/or substance use services.

Enforcement action by the Probation Service means the provision of warning letters and/or a return to court or custody, due to new offences or the violation of conditions. Recent (< 28 days) enforcement action was associated with deaths from external causes, both intentional and accidental, and identifies it as a risk marker specific to the CJS. The relationship was strongest for those on community sentences, in keeping with Borrill et al. (2017)’s study of 28 suspected suicides which emphasised those on community sentences rather than post-prison supervision as being at increased risk of suicide shortly after enforcement action had been initiated. This study cannot quantify whether this marker is due to the impact of the enforcement action or its potential outcomes (e.g., concerns over the outcome of returning to court or triggering a sense of unfairness, acute loss, hopelessness or loss of control as reported by those recalled to prison (Fitzalan Howard, 2019; Harris et al., 2020)). Based on available evidence it is likely to be an indicator of wider social, mental health or psychological issues, which are very common in this population (Power & McNally, 2022; Sirdifield, 2012), and are culminating in the violation of the terms of their sentence and the initiation of enforcement action. With enforcement action as a risk marker for suspected suicide, drug-related and accidental death, it will be important for justice, social, and health services to acknowledge the heightened risk when considering prioritisation for services which may prevent enforcement being required.

The network analysis identified that unemployment has a relationship with drug misuse and probation-identified suicide risk but not directly with any cause of death. This suggests that there may be a more complex relationship when considering the protective role of employment (Platt, et al., 2000), reflective of the more precarious employment situation for those in contact with the criminal justice (Nguyen et al., 2022) shown to increase the risk of suicide (Milner et al., 2018; Min et al., 2015). Furthermore, unilateral associations for perpetrators of familial violence or abuse with suspected suicide and drug overdose deaths reflected the emerging evidence of the relevance of the perpetration of family or intimate partner violence or abuse in suicide (Dewar et al., 2022; Kafka, et al., 2022) and emphasises the need for further research and practice review of this potential risk factor.

Although not significant in Gaussian Graphical model, some disproportionality within individual factors for minority groups was noted, which may reflect wider health and social inequalities e.g., females had higher relative rate of drug overdose, with disproportionate rates for homicide amongst people from Black and Mixed (lesser so, Asian) ethnic backgrounds (accounting for 29% of homicides). Other factors may have a mediating effect or the relatively small numbers in these groups may be affecting their power. Not withstanding the lack of power to detect such effects in the present data, this disproportionately is consistently reflected in official statistics across multiple years (Ministry of Justice, 2020) and in the general population with a 4-time risk of homicide within Black and ethnic minority populations (Office for National Statistics, 2022a, b) with further research required to focus further on these inequalities in context.

Probation services do not hold the same statutory duty of care as prisons hold for people in custody and relies on other statutory and partner agencies to deliver many key services to those under supervision (e.g., health and social care, housing, education). There are acknowledged challenges across many jurisdictions around information sharing, access, and user engagement with services (Her Majesty’s Inspectorate of Probation, 2022; Power & McNally, 2022; Prisons Probation Ombudsman, 2022). Further multi-agency examination of these deaths may provide evidence or learning for opportunities for prevention with these individuals who are in regular contact with the probation service.

## Limitations

This study has many strengths especially in its use of the full dataset for two years of all people who died under probation supervision. However, our study also has limitations.

The data in this study reflect the information as inputted by probation staff into a single system, and the data provided were not complete and may not reflect the full scope of available information on an individual. There are no structured assessments utilised by Probation for suicide risk and is reliant on professional judgement and information provided by other agencies (e.g. HM Prison Service or other agencies) All health data e.g., mental health problems or drug use, was reliant on information provided to, and recorded by, probation services either through self-report or by other agencies.

Most critically, the suspected cause of death is initially recorded by the probation service based on information provided at the time of death, which may not be the confirmed cause of death allocated by the coroners (Ministry of Justice, 2022b) and due to anonymous data, could not be further developed for this study. Nevertheless, collated data on suicide outcomes recorded at inquest between 2011–2021 have been cross-referenced with categorisations made by the Probation Service by Office for National Statistics (ONS) (2023); over 93% of suicides were accurately reported as self-inflicted death by Probation where a specific classification was provided. Approximately 25% of cases were not classified, resulting in a high number of exclusions from the analysis and we recommend that figures be viewed as trends rather than absolute rates. Future research would benefit from using data from confirmed causes of death and would allow for more nuanced analysis. The data for this study includes only those who have died and does not include those under supervision who did not die. This limits the application of findings as a risk model to the wider probation population. However, the comparative and multifactorial approach highlights clear differential factors which provide confidence in the findings.

## Conclusion

Our findings articulate the scale and unique characteristics and risk features, by cause of death, of people who die whilst under supervision in England and Wales. For the first time, the heightened risk of drug-related deaths, not only after custody release, but in the days and weeks after community sentencing, as well as the importance of enforcement action (due to a breach of probation conditions) inpremature deaths have been acknowledged.

The interlinking nature of suicide risk and drug use and the relevance of familial abuse on in premature deaths has been underlined, as well as recognising the potential health inequalities within this population and for certain minority groups. The understanding of deaths under supervision has been constrained by the limited research and data in this area. Nevertheless, there has been a gradual increase in focus on this population. This study emphasises the importance of joint working between health and justice, including reassessing an array of policies, training, and services to ensure they are responsive to the needs of those under probation supervision, and encompass those serving community sentences.

### Supplementary Information

**Additional file 1: Table A1.** Comparison of demographic information for included and excluded individuals from the sample

**Additional file 2: Table A2.** Partial Correlations (Posterior Mean) and 95% Posterior Probability Intervals (Credible Intervals; Cri) from the Gaussian Graphical Model of the Full Sample. **Table A3.** Partial Correlations (Posterior Mean) and 90 CI% Posterior Probability Intervals (Credible Intervals; CrI) from the Gaussian Graphical Model of the Post-Custody Release Population. **Table A4.** Partial Correlations (Posterior Mean) and 90% Posterior Probability Intervals (Credible Intervals; CrI) from the Gaussian Graphical Model of the Community Sentence Population.

## Data Availability

The data that support the findings of this study are available HM Prison and Probation Services but restrictions apply to the availability of these data, which were used under agreement for the current study, and so are not publicly available.
