# Supplementary material for: Deaths among adults under supervision of the England and Wales’ probation services: variation in individual and criminal justice-related factors by cause of death
Source: Health Justice. 2024 Feb 27;12:10. doi: 10.1186/s40352-024-00263-y (PMC10898034; doi:10.1186/s40352-024-00263-y)
Supplement: Supplementary file 1 — Additional file 1: Table A1. Comparison of demographic information for included and excluded individuals from the sample [file 40352_2024_263_MOESM1_ESM.docx]

**Additional File 1**

**Table A1.** Comparison of Demographic Information Included and Excluded Individuals

|  |  | Included | Excluded |  |  |
| --- | --- | --- | --- | --- | --- |
|  |  | *M* (SD) | *M* (SD) | *Difference* | *p* |
| Age |  | 46.0 (15.6) | 43.1 (12.4) | 2.93 | <.001 |
|  |  | *N* (%) | *N* (%) | *χ^2^* | *p* |
| Gender | Male | 1484 (87.3%) | 652 (87.2%) | 0.008 | .93 |
|  | Female | 216 (12.7%) | 96 (12.8%) |  |  |
| Ethnicity | Asian | 43 (2.6%) | 13 (1.8%) | 1.79 | .62 |
|  | Black | 53 (3.2%) | 24 (3.4%) |  |  |
|  | Mixed | 32 (1.9%) | 17 (2.4%) |  |  |
|  | White | 1535 (92.3%) | 658 (92.4%) |  |  |
| Sentence type | Post-custody release | 887 (52.2%) _+_ | 315 (42.2%) _-_ | 20.8 | <.001 |
|  | Community sentence | 813 (47.8%) _-_ | 432 (57.8%) _+_ |  |  |

Subscript “-“ indicates that a post-hoc test with *p*-values corrected using the False Discovery Rate showed that the cell count is significantly lower than expected within this group (row).

Subscript “+“ indicates that a post-hoc test with *p*-values corrected using the False Discovery Rate showed that the cell count is significantly higher than expected within this group (row).
