# Supplementary material for: Deaths among adults under supervision of the England and Wales’ probation services: variation in individual and criminal justice-related factors by cause of death
Source: Health Justice. 2024 Feb 27;12:10. doi: 10.1186/s40352-024-00263-y (PMC10898034; doi:10.1186/s40352-024-00263-y)
Supplement: Supplementary file 2 — Additional file 2: Table A2. Partial Correlations (Posterior Mean) and 95% Posterior Probability Intervals (Credible Intervals; Cri) from the Gaussian Graphical Model of the Full Sample. Table A3. Partial Correlations (Posterior Mean) and 90 CI% Posterior Probability Intervals (Credible Intervals; CrI) from the Gaussian Graphical Model of the Post-Custody Release Population. Table A4. Partial Correlations (Posterior Mean) and 90% Posterior Probability Intervals (Credible Intervals; CrI) from the Gaussian Graphical Model of the Community Sentence Population. [file 40352_2024_263_MOESM2_ESM.docx]

**Additional File 2**

| **Table S2.** Partial Correlations (Posterior Mean) and 95% Posterior Probability Intervals (Credible Intervals; Cri) from the Gaussian Graphical Model of the Full Sample between Causes of Death and Demographics, Health Information, Domestic Violence, and Other Risks | | | | | |
| --- | --- | --- | --- | --- | --- |
| **Domains** | **Variable 1** | **Variable 2** | **Partial**  **correlation** | **Upper**  **95% CrI** | **Lower**  **95% CrI** |
| Supervision | Post-custody | Drug overdose | -0.309 | -0.573 | 0.005 |
| type | Post-custody | Suspected suicide | -0.261 | -0.531 | 0.055 |
|  | Post-custody | Accidental | -0.297 | -0.554 | 0.015 |
|  | Post-custody | Homicide | **-0.369** | **-0.635** | **-0.039** |
|  | Post-custody | Female sex | -0.135 | -0.419 | 0.140 |
|  | Post-custody | Ethnic minority | -0.111 | -0.366 | 0.139 |
|  | Post-custody | Muslim | 0.255 | -0.042 | 0.555 |
|  | Post-custody | Non settled acc. | -0.014 | -0.236 | 0.184 |
|  | Post-custody | Unemployment | 0.073 | -0.166 | 0.311 |
|  | Post-custody | Enforcement | 0.085 | -0.128 | 0.313 |
|  | Post-custody | Drug misuse | **0.443** | **0.230** | **0.661** |
|  | Post-custody | Known suicide risk | 0.010 | -0.195 | 0.212 |
|  | Post-custody | Mental health condition | 0.012 | -0.177 | 0.243 |
|  | Post-custody | High/Very high RoSH | 0.128 | -0.08 | 0.354 |
|  | Post-custody | DV perpetration | -0.031 | -0.261 | 0.212 |
|  | Post-custody | DV victimisation | 0.031 | -0.260 | 0.293 |
| Cause | Drug overdose | Suspected suicide | **-0.827** | **-0.912** | **-0.682** |
| of death | Drug overdose | Accidental | **-0.823** | **-0.908** | **-0.713** |
|  | Drug overdose | Homicide | **-0.721** | **-0.850** | **-0.514** |
|  | Drug overdose | Female sex | 0.096 | -0.364 | 0.481 |
|  | Drug overdose | Ethnic minority | 0.019 | -0.435 | 0.393 |
|  | Drug overdose | Muslim | 0.141 | -0.277 | 0.534 |
|  | Drug overdose | Non settled acc. | 0.074 | -0.271 | 0.358 |
|  | Drug overdose | Unemployment | -0.188 | -0.588 | 0.180 |
|  | Drug overdose | Enforcement | **0.437** | **0.131** | **0.694** |
|  | Drug overdose | Drug misuse | **0.647** | **0.377** | **0.839** |
|  | Drug overdose | Known suicide risk | **0.344** | **0.053** | **0.604** |
|  | Drug overdose | Mental health condition | 0.29 | -0.014 | 0.574 |
|  | Drug overdose | High/Very high RoSH | 0.138 | -0.231 | 0.504 |
|  | Drug overdose | DV perpetration | 0.263 | -0.026 | 0.588 |
|  | Drug overdose | DV victimisation | -0.103 | -0.496 | 0.316 |
|  | Suspected suicide | Homicide | **-0.599** | **-0.775** | **-0.304** |
|  | Suspected suicide | Accidental | **-0.744** | **-0.867** | **-0.58** |
|  | Suspected suicide | Female sex | -0.019 | -0.449 | 0.342 |
|  | Suspected suicide | Ethnic minority | 0.079 | -0.335 | 0.434 |
|  | Suspected suicide | Muslim | -0.025 | -0.431 | 0.363 |
|  | Suspected suicide | Non settled acc. | 0.099 | -0.221 | 0.366 |
|  | Suspected suicide | Unemployment | -0.324 | -0.698 | 0.016 |
|  | Suspected suicide | Enforcement | **0.409** | **0.116** | **0.656** |
|  | Suspected suicide | Drug misuse | **0.546** | **0.228** | **0.791** |
|  | Suspected suicide | Known suicide risk | **0.461** | **0.211** | **0.694** |
|  | Suspected suicide | Mental health condition | 0.212 | -0.083 | 0.512 |
|  | Suspected suicide | High/Very high RoSH | 0.162 | -0.187 | 0.522 |
|  | Suspected suicide | DV perpetration | 0.165 | -0.126 | 0.475 |
|  | Suspected suicide | DV victimisation | 0.003 | -0.376 | 0.406 |
|  | Accidental | Homicide | **-0.627** | **-0.789** | **-0.396** |
|  | Accidental | Female sex | 0.059 | -0.404 | 0.476 |
|  | Accidental | Ethnic minority | -0.016 | -0.485 | 0.376 |
|  | Accidental | Muslim | 0.152 | -0.229 | 0.567 |
|  | Accidental | Non settled acc. | 0.008 | -0.327 | 0.290 |
|  | Accidental | Unemployment | -0.276 | -0.586 | 0.077 |
|  | Accidental | Enforcement | **0.427** | **0.133** | **0.679** |
|  | Accidental | Drug misuse | **0.514** | **0.196** | **0.749** |
|  | Accidental | Known suicide risk | **0.325** | **0.056** | **0.565** |
|  | Accidental | Mental health condition | 0.264 | -0.039 | 0.541 |
|  | Accidental | High/Very high RoSH | 0.185 | -0.175 | 0.508 |
|  | Accidental | DV perpetration | 0.219 | -0.096 | 0.567 |
|  | Accidental | DV victimisation | -0.071 | -0.483 | 0.386 |
|  | Homicide | Female sex | 0.023 | -0.392 | 0.410 |
|  | Homicide | Ethnic minority | 0.111 | -0.256 | 0.455 |
|  | Homicide | Muslim | 0.328 | -0.061 | 0.645 |
|  | Homicide | Non settled acc. | 0.083 | -0.263 | 0.357 |
|  | Homicide | Unemployment | -0.130 | -0.462 | 0.194 |
|  | Homicide | Enforcement | **0.363** | **0.055** | **0.615** |
|  | Homicide | Drug misuse | **0.561** | **0.267** | **0.768** |
|  | Homicide | Known suicide risk | 0.201 | -0.101 | 0.483 |
|  | Homicide | Mental health condition | 0.242 | -0.075 | 0.529 |
|  | Homicide | High/Very high RoSH | 0.025 | -0.33 | 0.368 |
|  | Homicide | DV perpetration | 0.240 | -0.050 | 0.540 |
|  | Homicide | DV victimisation | -0.113 | -0.502 | 0.319 |
| Demographics | Female sex | Ethnic minority | -0.075 | -0.404 | 0.317 |
|  | Female sex | Muslim | -0.031 | -0.476 | 0.371 |
|  | Female sex | Non settled acc. | 0.107 | -0.184 | 0.369 |
|  | Female sex | Unemployment | -0.126 | -0.456 | 0.226 |
|  | Female sex | Enforcement | 0.026 | -0.282 | 0.339 |
|  | Female sex | Drug misuse | 0.163 | -0.205 | 0.499 |
|  | Female sex | Known suicide risk | 0.072 | -0.223 | 0.364 |
|  | Female sex | Mental health condition | 0.004 | -0.349 | 0.324 |
|  | Female sex | High/Very high RoSH | -0.203 | -0.512 | 0.088 |
|  | Female sex | DV perpetration | **-0.604** | **-0.758** | **-0.404** |
|  | Female sex | DV victimisation | **0.777** | **0.647** | **0.865** |
|  | Ethnic minority | Muslim | **0.566** | **0.353** | **0.740** |
|  | Ethnic minority | Non settled acc. | 0.022 | -0.215 | 0.278 |
|  | Ethnic minority | Unemployment | 0.047 | -0.228 | 0.335 |
|  | Ethnic minority | Enforcement | -0.153 | -0.385 | 0.184 |
|  | Ethnic minority | Drug misuse | -0.049 | -0.378 | 0.299 |
|  | Ethnic minority | Known suicide risk | -0.033 | -0.272 | 0.209 |
|  | Ethnic minority | Mental health condition | -0.124 | -0.345 | 0.141 |
|  | Ethnic minority | High/Very high RoSH | -0.044 | -0.309 | 0.229 |
|  | Ethnic minority | DV perpetration | -0.020 | -0.339 | 0.313 |
|  | Ethnic minority | DV victimisation | 0.067 | -0.272 | 0.414 |
|  | Muslim | Non settled acc. | 0.032 | -0.309 | 0.353 |
|  | Muslim | Unemployment | -0.141 | -0.457 | 0.168 |
|  | Muslim | Enforcement | 0.066 | -0.294 | 0.373 |
|  | Muslim | Drug misuse | -0.138 | -0.494 | 0.232 |
|  | Muslim | Known suicide risk | 0.110 | -0.192 | 0.405 |
|  | Muslim | Mental health condition | -0.058 | -0.362 | 0.253 |
|  | Muslim | High/Very high RoSH | 0.149 | -0.176 | 0.449 |
|  | Muslim | DV perpetration | -0.190 | -0.571 | 0.154 |
|  | Muslim | DV victimisation | 0.105 | -0.263 | 0.542 |
| Personal | Non settled acc. | Unemployment | 0.002 | -0.203 | 0.221 |
| circumstances | Non settled acc. | Enforcement | 0.064 | -0.123 | 0.265 |
|  | Non settled acc. | Drug misuse | -0.104 | -0.327 | 0.192 |
|  | Non settled acc. | Known suicide risk | -0.089 | -0.271 | 0.098 |
|  | Non settled acc. | Mental health condition | 0.066 | -0.101 | 0.269 |
|  | Non settled acc. | High/Very high RoSH | **0.180** | **0.005** | **0.349** |
|  | Non settled acc. | DV perpetration | 0.034 | -0.209 | 0.275 |
|  | Non settled acc. | DV victimisation | -0.162 | -0.438 | 0.148 |
|  | Unemployment | Enforcement | 0.174 | -0.084 | 0.419 |
|  | Unemployment | Drug misuse | 0.254 | -0.046 | 0.602 |
|  | Unemployment | Known suicide risk | **0.308** | **0.068** | **0.616** |
|  | Unemployment | Mental health condition | 0.132 | -0.138 | 0.430 |
|  | Unemployment | High/Very high RoSH | 0.179 | -0.168 | 0.521 |
|  | Unemployment | DV perpetration | -0.140 | -0.413 | 0.157 |
|  | Unemployment | DV victimisation | 0.115 | -0.203 | 0.434 |
| Offence | Enforcement | Drug misuse | **-0.271** | **-0.548** | **-0.011** |
| information | Enforcement | Known suicide risk | -0.170 | -0.403 | 0.029 |
|  | Enforcement | Mental health condition | -0.145 | -0.362 | 0.054 |
|  | Enforcement | High/Very high RoSH | -0.162 | -0.398 | 0.088 |
|  | Enforcement | DV perpetration | -0.019 | -0.300 | 0.235 |
|  | Enforcement | DV victimisation | 0.078 | -0.249 | 0.373 |
| Risk factors | Drug misuse | Known suicide risk | -0.177 | -0.509 | 0.075 |
|  | Drug misuse | Mental health condition | -0.152 | -0.436 | 0.109 |
|  | Drug misuse | High/Very high RoSH | 0.042 | -0.393 | 0.318 |
|  | Drug misuse | DV perpetration | 0.004 | -0.303 | 0.276 |
|  | Drug misuse | DV victimisation | -0.116 | -0.463 | 0.247 |
|  | Known suicide risk | Mental health condition | 0.099 | -0.130 | 0.297 |
|  | Known suicide risk | High/Very high RoSH | -0.059 | -0.342 | 0.166 |
|  | Known suicide risk | DV perpetration | 0.081 | -0.189 | 0.323 |
|  | Known suicide risk | DV victimisation | 0.009 | -0.312 | 0.300 |
|  | Mental health condition | High/Very high RoSH | -0.001 | -0.269 | 0.202 |
|  | Mental health condition | DV perpetration | -0.044 | -0.305 | 0.187 |
|  | Mental health condition | DV victimisation | 0.188 | -0.135 | 0.509 |
|  | High/Very high RoSH | DV perpetration | 0.092 | -0.205 | 0.361 |
|  | High/Very high RoSH | DV victimisation | 0.067 | -0.264 | 0.397 |
|  | DV perpetration | DV victimisation | **0.605** | **0.372** | **0.755** |

| **Table A3.** Partial Correlations (Posterior Mean) and 90 CI% Posterior Probability Intervals (Credible Intervals; CrI) from the Gaussian Graphical Model of the Post-Custody Release Population between Causes of Death and Demographics, Health Information, Domestic Violence, and Other Risks | | | | | |
| --- | --- | --- | --- | --- | --- |
| Domains | Variable 1 | Variable 2 | Partial correlaction | Upper 90% CrI | Lower 90% CrI |
| Cause | Drug overdose | Suspected suicide | **-0.732** | **-0.86** | **-0.556** |
| of death | Drug overdose | Accidental | **-0.721** | **-0.864** | **-0.564** |
|  | Drug overdose | Homicide | **-0.502** | **-0.758** | **-0.196** |
|  | Drug overdose | Female sex | 0.090 | -0.311 | 0.441 |
|  | Drug overdose | Ethnic minority | 0.005 | -0.357 | 0.34 |
|  | Drug overdose | Muslim | 0.080 | -0.247 | 0.446 |
|  | Drug overdose | Non settled acc. | 0.165 | -0.169 | 0.463 |
|  | Drug overdose | Unemployment | -0.122 | -0.483 | 0.251 |
|  | Drug overdose | Enforcement | 0.298 | -0.019 | 0.621 |
|  | Drug overdose | Drug misuse | **0.585** | **0.339** | **0.779** |
|  | Drug overdose | Known suicide risk | 0.211 | -0.094 | 0.497 |
|  | Drug overdose | Mental health condition | 0.295 | -0.002 | 0.549 |
|  | Drug overdose | High/Very high RoSH | 0.012 | -0.273 | 0.303 |
|  | Drug overdose | DV perpetration | 0.232 | -0.063 | 0.508 |
|  | Drug overdose | DV victimisation | 0.009 | -0.418 | 0.379 |
|  | Suspected suicide | Accidental | **-0.558** | **-0.765** | **-0.312** |
|  | Suspected suicide | Homicide | -0.304 | -0.642 | 0.078 |
|  | Suspected suicide | Female sex | -0.001 | -0.386 | 0.371 |
|  | Suspected suicide | Ethnic minority | -0.040 | -0.389 | 0.303 |
|  | Suspected suicide | Muslim | -0.080 | -0.430 | 0.300 |
|  | Suspected suicide | Non settled acc. | 0.120 | -0.178 | 0.424 |
|  | Suspected suicide | Unemployment | -0.308 | -0.595 | 0.053 |
|  | Suspected suicide | Enforcement | 0.279 | -0.044 | 0.566 |
|  | Suspected suicide | Drug misuse | **0.392** | **0.098** | **0.666** |
|  | Suspected suicide | Known suicide risk | **0.364** | **0.093** | **0.600** |
|  | Suspected suicide | Mental health condition | 0.218 | -0.093 | 0.484 |
|  | Suspected suicide | High/Very high RoSH | 0.126 | -0.161 | 0.424 |
|  | Suspected suicide | DV perpetration | 0.074 | -0.241 | 0.372 |
|  | Suspected suicide | DV victimisation | 0.115 | -0.294 | 0.511 |
|  | Accidental | Homicide | -0.322 | -0.683 | 0.018 |
|  | Accidental | Female sex | 0.122 | -0.246 | 0.455 |
|  | Accidental | Ethnic minority | -0.022 | -0.351 | 0.322 |
|  | Accidental | Muslim | 0.086 | -0.264 | 0.430 |
|  | Accidental | Non settled acc. | 0.134 | -0.196 | 0.426 |
|  | Accidental | Unemployment | -0.048 | -0.443 | 0.359 |
|  | Accidental | Enforcement | 0.221 | -0.121 | 0.577 |
|  | Accidental | Drug misuse | **0.401** | **0.154** | **0.649** |
|  | Accidental | Known suicide risk | 0.176 | -0.130 | 0.459 |
|  | Accidental | Mental health condition | 0.223 | -0.057 | 0.488 |
|  | Accidental | High/Very high RoSH | -0.010 | -0.283 | 0.269 |
|  | Accidental | DV perpetration | 0.187 | -0.136 | 0.440 |
|  | Accidental | DV victimisation | 0.067 | -0.348 | 0.464 |
|  | Homicide | Female sex | -0.146 | -0.608 | 0.313 |
|  | Homicide | Ethnic minority | 0.242 | -0.119 | 0.559 |
|  | Homicide | Muslim | 0.275 | -0.107 | 0.646 |
|  | Homicide | Non settled acc. | 0.097 | -0.291 | 0.455 |
|  | Homicide | Unemployment | -0.056 | -0.438 | 0.314 |
|  | Homicide | Enforcement | 0.243 | -0.073 | 0.557 |
|  | Homicide | Drug misuse | 0.327 | -0.008 | 0.662 |
|  | Homicide | Known suicide risk | 0.096 | -0.233 | 0.422 |
|  | Homicide | Mental health condition | 0.280 | -0.074 | 0.615 |
|  | Homicide | High/Very high RoSH | -0.050 | -0.393 | 0.283 |
|  | Homicide | DV perpetration | 0.173 | -0.172 | 0.473 |
|  | Homicide | DV victimisation | -0.142 | -0.589 | 0.294 |
| Demographics | Female sex | Ethnic minority | -0.127 | -0.481 | 0.290 |
|  | Female sex | Muslim | 0.114 | -0.286 | 0.524 |
|  | Female sex | Non settled acc. | 0.032 | -0.264 | 0.329 |
|  | Female sex | Unemployment | -0.152 | -0.481 | 0.262 |
|  | Female sex | Enforcement | 0.040 | -0.257 | 0.317 |
|  | Female sex | Drug misuse | 0.188 | -0.128 | 0.498 |
|  | Female sex | Known suicide risk | 0.154 | -0.151 | 0.422 |
|  | Female sex | Mental health condition | 0.046 | -0.283 | 0.391 |
|  | Female sex | High/Very high RoSH | -0.071 | -0.396 | 0.268 |
|  | Female sex | DV perpetration | **-0.464** | **-0.690** | **-0.221** |
|  | Female sex | DV victimisation | **0.527** | **0.243** | **0.759** |
|  | Ethnic minority | Muslim | **0.488** | **0.237** | **0.686** |
|  | Ethnic minority | Non settled acc. | -0.054 | -0.376 | 0.258 |
|  | Ethnic minority | Unemployment | 0.001 | -0.327 | 0.352 |
|  | Ethnic minority | Enforcement | -0.091 | -0.406 | 0.209 |
|  | Ethnic minority | Drug misuse | -0.001 | -0.336 | 0.341 |
|  | Ethnic minority | Known suicide risk | 0.177 | -0.110 | 0.451 |
|  | Ethnic minority | Mental health condition | -0.084 | -0.39 | 0.226 |
|  | Ethnic minority | High/Very high RoSH | -0.007 | -0.329 | 0.255 |
|  | Ethnic minority | DV perpetration | -0.179 | -0.442 | 0.118 |
|  | Ethnic minority | DV victimisation | 0.068 | -0.406 | 0.513 |
|  | Muslim | Non settled acc. | -0.003 | -0.341 | 0.335 |
|  | Muslim | Unemployment | -0.156 | -0.471 | 0.187 |
|  | Muslim | Enforcement | 0.064 | -0.274 | 0.422 |
|  | Muslim | Drug misuse | -0.100 | -0.470 | 0.299 |
|  | Muslim | Known suicide risk | 0.029 | -0.322 | 0.357 |
|  | Muslim | Mental health condition | -0.012 | -0.368 | 0.355 |
|  | Muslim | High/Very high RoSH | 0.202 | -0.116 | 0.525 |
|  | Muslim | DV perpetration | -0.138 | -0.507 | 0.233 |
|  | Muslim | DV victimisation | 0.084 | -0.409 | 0.556 |
| Personal | Non settled acc. | Unemployment | -0.179 | -0.457 | 0.124 |
| circumstances | Non settled acc. | Enforcement | 0.136 | -0.145 | 0.385 |
|  | Non settled acc. | Drug misuse | -0.179 | -0.447 | 0.074 |
|  | Non settled acc. | Known suicide risk | 0.139 | -0.128 | 0.382 |
|  | Non settled acc. | Mental health condition | 0.115 | -0.131 | 0.387 |
|  | Non settled acc. | High/Very high RoSH | 0.086 | -0.138 | 0.296 |
|  | Non settled acc. | DV perpetration | -0.092 | -0.362 | 0.144 |
|  | Non settled acc. | DV victimisation | **-0.317** | **-0.582** | **-0.023** |
|  | Unemployment | Enforcement | 0.204 | -0.136 | 0.515 |
|  | Unemployment | Drug misuse | 0.082 | -0.258 | 0.434 |
|  | Unemployment | Known suicide risk | **0.385** | **0.083** | **0.705** |
|  | Unemployment | Mental health condition | 0.133 | -0.189 | 0.453 |
|  | Unemployment | High/Very high RoSH | 0.186 | -0.136 | 0.487 |
|  | Unemployment | DV perpetration | -0.247 | -0.554 | 0.050 |
|  | Unemployment | DV victimisation | 0.063 | -0.409 | 0.501 |
| Offence | Enforcement | Drug misuse | -0.120 | -0.433 | 0.153 |
| information | Enforcement | Known suicide risk | -0.129 | -0.355 | 0.103 |
|  | Enforcement | Mental health condition | -0.168 | -0.432 | 0.043 |
|  | Enforcement | High/Very high RoSH | -0.086 | -0.344 | 0.151 |
|  | Enforcement | DV perpetration | 0.104 | -0.177 | 0.378 |
|  | Enforcement | DV victimisation | 0.178 | -0.178 | 0.512 |
| Risk factors | Drug misuse | Known suicide risk | -0.006 | -0.311 | 0.294 |
|  | Drug misuse | Mental health condition | -0.018 | -0.294 | 0.243 |
|  | Drug misuse | High/Very high RoSH | 0.156 | -0.094 | 0.386 |
|  | Drug misuse | DV perpetration | -0.021 | -0.322 | 0.275 |
|  | Drug misuse | DV victimisation | -0.264 | -0.542 | 0.079 |
|  | Known suicide risk | Mental health condition | 0.019 | -0.263 | 0.269 |
|  | Known suicide risk | High/Very high RoSH | -0.086 | -0.312 | 0.124 |
|  | Known suicide risk | DV perpetration | **0.287** | **0.029** | **0.519** |
|  | Known suicide risk | DV victimisation | 0.066 | -0.285 | 0.401 |
|  | Mental health condition | High/Very high RoSH | 0.020 | -0.230 | 0.266 |
|  | Mental health condition | DV perpetration | 0.066 | -0.205 | 0.329 |
|  | Mental health condition | DV victimisation | 0.263 | -0.103 | 0.626 |
|  | High/Very high RoSH | DV perpetration | 0.258 | -0.005 | 0.539 |
|  | High/Very high RoSH | DV victimisation | -0.029 | -0.402 | 0.333 |
|  | DV perpetration | DV victimisation | 0.265 | -0.064 | 0.583 |

| **Table A4:** Partial Correlations (Posterior Mean) and 90% Posterior Probability Intervals (Credible Intervals; CrI) from the Gaussian Graphical Model of the Community Sentence Population between Causes of Death and Demographics, Health Information, Domestic Violence, and Other Risks | | | | | |
| --- | --- | --- | --- | --- | --- |
| Domain | Variable 1 | Variable 2 | Partial correlaction | Upper 90% CrI | Lower 90% CrI |
| Cause | Drug overdose | Suspected suicide | **-0.761** | **-0.885** | **-0.599** |
| of death | Drug overdose | Accidental | **-0.708** | **-0.848** | **-0.506** |
|  | Drug overdose | Homicide | **-0.589** | **-0.782** | **-0.331** |
|  | Drug overdose | Female sex | -0.038 | -0.393 | 0.356 |
|  | Drug overdose | Ethnic minority | 0.017 | -0.355 | 0.432 |
|  | Drug overdose | Muslim | -0.002 | -0.403 | 0.404 |
|  | Drug overdose | Non settled acc. | 0.040 | -0.332 | 0.459 |
|  | Drug overdose | Unemployment | -0.156 | -0.573 | 0.231 |
|  | Drug overdose | Enforcement | **0.369** | **0.063** | **0.632** |
|  | Drug overdose | Drug misuse | **0.433** | **0.136** | **0.712** |
|  | Drug overdose | Known suicide risk | 0.333 | -0.038 | 0.628 |
|  | Drug overdose | Mental health condition | 0.119 | -0.264 | 0.483 |
|  | Drug overdose | High/Very high RoSH | 0.081 | -0.298 | 0.472 |
|  | Drug overdose | DV perpetration | 0.107 | -0.221 | 0.473 |
|  | Drug overdose | DV victimisation | -0.008 | -0.439 | 0.362 |
|  | Suspected suicide | Accidental | **-0.652** | **-0.818** | **-0.444** |
|  | Suspected suicide | Homicide | **-0.490** | **-0.738** | **-0.200** |
|  | Suspected suicide | Female sex | -0.090 | -0.429 | 0.277 |
|  | Suspected suicide | Ethnic minority | 0.069 | -0.340 | 0.452 |
|  | Suspected suicide | Muslim | -0.070 | -0.474 | 0.367 |
|  | Suspected suicide | Non settled acc. | 0.104 | -0.241 | 0.463 |
|  | Suspected suicide | Unemployment | -0.307 | -0.684 | 0.058 |
|  | Suspected suicide | Enforcement | **0.309** | **0.009** | **0.600** |
|  | Suspected suicide | Drug misuse | **0.377** | **0.072** | **0.699** |
|  | Suspected suicide | Known suicide risk | **0.461** | **0.143** | **0.717** |
|  | Suspected suicide | Mental health condition | 0.029 | -0.351 | 0.412 |
|  | Suspected suicide | High/Very high RoSH | 0.043 | -0.305 | 0.416 |
|  | Suspected suicide | DV perpetration | 0.127 | -0.182 | 0.432 |
|  | Suspected suicide | DV victimisation | -0.009 | -0.411 | 0.346 |
|  | Accidental | Homicide | **-0.459** | **-0.720** | **-0.131** |
|  | Accidental | Female sex | -0.114 | -0.474 | 0.256 |
|  | Accidental | Ethnic minority | -0.038 | -0.403 | 0.379 |
|  | Accidental | Muslim | 0.014 | -0.383 | 0.407 |
|  | Accidental | Non settled acc. | -0.085 | -0.433 | 0.274 |
|  | Accidental | Unemployment | -0.310 | -0.635 | 0.033 |
|  | Accidental | Enforcement | **0.396** | **0.111** | **0.638** |
|  | Accidental | Drug misuse | 0.266 | -0.086 | 0.575 |
|  | Accidental | Known suicide risk | 0.227 | -0.105 | 0.522 |
|  | Accidental | Mental health condition | 0.110 | -0.232 | 0.427 |
|  | Accidental | High/Very high RoSH | 0.214 | -0.134 | 0.564 |
|  | Accidental | DV perpetration | 0.069 | -0.274 | 0.405 |
|  | Accidental | DV victimisation | 0.040 | -0.373 | 0.390 |
|  | Homicide | Female sex | -0.045 | -0.395 | 0.334 |
|  | Homicide | Ethnic minority | -0.043 | -0.504 | 0.391 |
|  | Homicide | Muslim | 0.375 | -0.035 | 0.733 |
|  | Homicide | Non settled acc. | 0.035 | -0.328 | 0.494 |
|  | Homicide | Unemployment | -0.110 | -0.506 | 0.313 |
|  | Homicide | Enforcement | 0.247 | -0.041 | 0.528 |
|  | Homicide | Drug misuse | 0.406 | -0.003 | 0.716 |
|  | Homicide | Known suicide risk | 0.176 | -0.243 | 0.564 |
|  | Homicide | Mental health condition | 0.149 | -0.265 | 0.483 |
|  | Homicide | High/Very high RoSH | -0.082 | -0.447 | 0.287 |
|  | Homicide | DV perpetration | 0.141 | -0.232 | 0.498 |
|  | Homicide | DV victimisation | -0.063 | -0.415 | 0.352 |
| Demographics | Female sex | Ethnic minority | -0.132 | -0.523 | 0.254 |
|  | Female sex | Muslim | 0.033 | -0.355 | 0.524 |
|  | Female sex | Non settled acc. | 0.056 | -0.32 | 0.391 |
|  | Female sex | Unemployment | -0.036 | -0.392 | 0.311 |
|  | Female sex | Enforcement | 0.124 | -0.193 | 0.425 |
|  | Female sex | Drug misuse | 0.067 | -0.264 | 0.380 |
|  | Female sex | Known suicide risk | 0.053 | -0.328 | 0.382 |
|  | Female sex | Mental health condition | 0.021 | -0.316 | 0.321 |
|  | Female sex | High/Very high RoSH | -0.300 | -0.608 | 0.073 |
|  | Female sex | DV perpetration | **-0.591** | **-0.759** | **-0.387** |
|  | Female sex | DV victimisation | **0.779** | **0.624** | **0.891** |
|  | Ethnic minority | Muslim | **0.657** | **0.348** | **0.856** |
|  | Ethnic minority | Non settled acc. | -0.040 | -0.412 | 0.326 |
|  | Ethnic minority | Unemployment | 0.178 | -0.167 | 0.556 |
|  | Ethnic minority | Enforcement | -0.220 | -0.507 | 0.063 |
|  | Ethnic minority | Drug misuse | -0.213 | -0.551 | 0.166 |
|  | Ethnic minority | Known suicide risk | -0.226 | -0.569 | 0.097 |
|  | Ethnic minority | Mental health condition | -0.105 | -0.436 | 0.224 |
|  | Ethnic minority | High/Very high RoSH | -0.102 | -0.445 | 0.232 |
|  | Ethnic minority | DV perpetration | 0.012 | -0.390 | 0.395 |
|  | Ethnic minority | DV victimisation | 0.158 | -0.248 | 0.618 |
|  | Muslim | Non settled acc. | 0.172 | -0.247 | 0.555 |
|  | Muslim | Unemployment | -0.216 | -0.609 | 0.183 |
|  | Muslim | Enforcement | 0.177 | -0.156 | 0.476 |
|  | Muslim | Drug misuse | 0.027 | -0.459 | 0.443 |
|  | Muslim | Known suicide risk | 0.225 | -0.184 | 0.613 |
|  | Muslim | Mental health condition | -0.146 | -0.520 | 0.265 |
|  | Muslim | High/Very high RoSH | 0.117 | -0.277 | 0.477 |
|  | Muslim | DV perpetration | -0.071 | -0.482 | 0.329 |
|  | Muslim | DV victimisation | -0.016 | -0.532 | 0.403 |
| Personal | Non settled acc. | Unemployment | 0.128 | -0.185 | 0.421 |
| circumstances | Non settled acc. | Enforcement | 0.070 | -0.226 | 0.331 |
|  | Non settled acc. | Drug misuse | -0.166 | -0.467 | 0.119 |
|  | Non settled acc. | Known suicide risk | **-0.286** | **-0.521** | **-0.025** |
|  | Non settled acc. | Mental health condition | 0.078 | -0.158 | 0.312 |
|  | Non settled acc. | High/Very high RoSH | **0.360** | **0.100** | **0.590** |
|  | Non settled acc. | DV perpetration | -0.085 | -0.395 | 0.214 |
|  | Non settled acc. | DV victimisation | 0.012 | -0.344 | 0.389 |
|  | Unemployment | Enforcement | 0.145 | -0.112 | 0.442 |
|  | Unemployment | Drug misuse | **0.421** | **0.081** | **0.723** |
|  | Unemployment | Known suicide risk | **0.298** | **0.003** | **0.603** |
|  | Unemployment | Mental health condition | 0.105 | -0.190 | 0.399 |
|  | Unemployment | High/Very high RoSH | 0.143 | -0.192 | 0.488 |
|  | Unemployment | DV perpetration | -0.021 | -0.352 | 0.294 |
|  | Unemployment | DV victimisation | -0.022 | -0.443 | 0.360 |
| Offence | Enforcement | Drug misuse | -0.173 | -0.461 | 0.098 |
| information | Enforcement | Known suicide risk | -0.146 | -0.416 | 0.131 |
|  | Enforcement | Mental health condition | -0.053 | -0.321 | 0.194 |
|  | Enforcement | High/Very high RoSH | -0.178 | -0.448 | 0.102 |
|  | Enforcement | DV perpetration | 0.062 | -0.234 | 0.319 |
|  | Enforcement | DV victimisation | -0.034 | -0.360 | 0.307 |
| Risk factors | Drug misuse | Known suicide risk | **-0.263** | **-0.559** | **-0.002** |
|  | Drug misuse | Mental health condition | -0.186 | -0.480 | 0.123 |
|  | Drug misuse | High/Very high RoSH | 0.070 | -0.251 | 0.388 |
|  | Drug misuse | DV perpetration | 0.023 | -0.308 | 0.310 |
|  | Drug misuse | DV victimisation | 0.063 | -0.294 | 0.443 |
|  | Known suicide risk | Mental health condition | 0.193 | -0.122 | 0.446 |
|  | Known suicide risk | High/Very high RoSH | 0.098 | -0.241 | 0.390 |
|  | Known suicide risk | DV perpetration | -0.008 | -0.328 | 0.291 |
|  | Known suicide risk | DV victimisation | 0.079 | -0.281 | 0.508 |
|  | Mental health condition | High/Very high RoSH | 0.012 | -0.250 | 0.286 |
|  | Mental health condition | DV perpetration | -0.029 | -0.316 | 0.253 |
|  | Mental health condition | DV victimisation | 0.175 | -0.162 | 0.530 |
|  | High/Very high RoSH | DV perpetration | 0.050 | -0.265 | 0.340 |
|  | High/Very high RoSH | DV victimisation | 0.076 | -0.311 | 0.487 |
|  | DV perpetration | DV victimisation | **0.647** | **0.459** | **0.794** |
